# Supplementary material for: Two-photon lithography-fabricated deterministic lateral displacement microfluidic system for efficient minicell purification in cancer therapy
Source: Biomed Microdevices. 2025 Sep 20;27(4):42. doi: 10.1007/s10544-025-00774-x (PMC12450229; doi:10.1007/s10544-025-00774-x)
Supplement: Supplementary file 1 — Supplementary Material 1 (DOCX 17.3 MB) [file 10544_2025_774_MOESM1_ESM.docx]

Supplementary Information (SI)


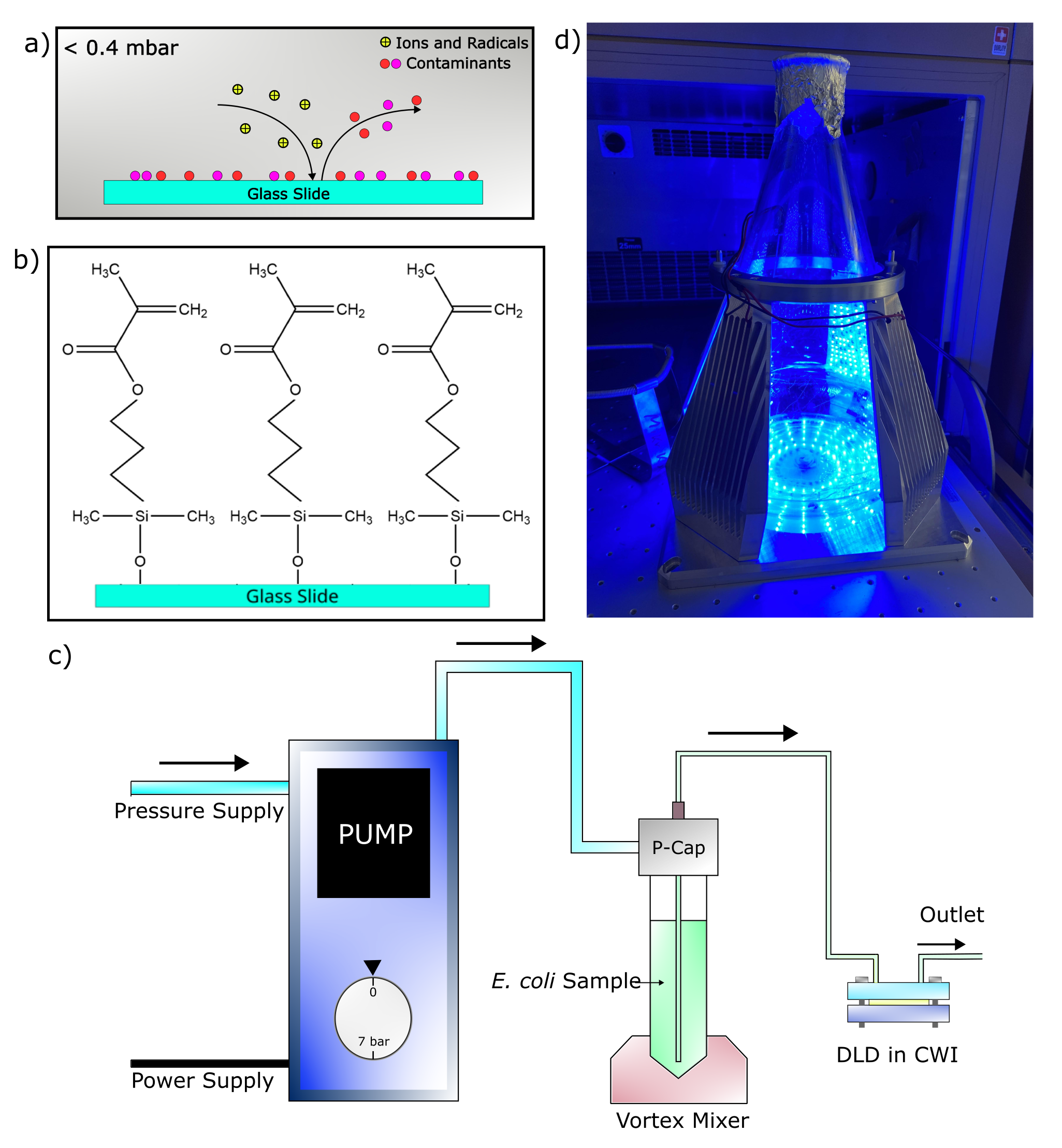


**Figure S1.** a) Plasma cleaning of the glass slide b) MACS functionalization of the glass slide c) Schematic of minicell purification experimental setup d) custom-built LED device to trigger minicell formation

The surface of the glass slide is initially plasma cleaned (**Figure S1a)** and then functionalized with MACS (**Figure S1b)** before TPL printing. Following TPL printing, to develop microchannels and run the experiments, isopropanol is passed with an inlet pressure of 7 bar through the channels with a setup as seen in (**Figure S1c)**. A CWI ensures a seamless, airtight connection between the microfluidic chip and external tubing, preventing air gaps or leaks that could disrupt the process as seen in. This setup enables effective high-pressure flushing, eliminating undeveloped resin and ensuring unobstructed microchannels for optimal performance.

**Table S1**. DLD parameters and resulting critical diameters

| Downstream Post Gap  G_D_  (µm) | Lateral Post Gap  G_L_  (µm) | Shift Angle  θ  (°) | Post Diameter  D_p_  (µm) | Channel Length  L  (mm) | Critical Diameter  D_C_  (µm) |
| --- | --- | --- | --- | --- | --- |
| 7 | 7 | 0.75 | 25 | 19 | 1.223 |
| 6 | 6 | 0.75 | 25 | 19 | 1.048 |
| 5 | 5 | 0. 75 | 25 | 19 | 0.873 |
| 4 | 4 | 0.75 | 25 | 19 | 0.699 |
| 4 | 5 | 0.75 | 25 | 19 | <0.699 |
| 4 | 6 | 0.75 | 25 | 19 | <0.699 |
| 4 | 7 | 0.75 | 25 | 19 | <0.699 |
| 3 | 4 | 0.75 | 25 | 19 | 0.524 |
| 2 | 4 | 0.75 | 25 | 19 | 0.349 |
